# Evidence of constant diversification punctuated by a mass extinction in the African cycads

**DOI:** 10.1002/ece3.880

**Published:** 2013-12-11

**Authors:** Kowiyou Yessoufou, Samuel O Bamigboye, Barnabas H Daru, Michelle van der Bank

**Affiliations:** African Centre for DNA Barcoding, Department of Botany and Plant Biotechnology, University of JohannesburgP. O. Box 524, Auckland Park, Johannesburg, 2006, South Africa

**Keywords:** Climate change, *Encephalartos*, extinction, gymnosperms, adaptive radiation, subtropical Africa.

## Abstract

The recent evidence that extant cycads are not living fossils triggered a renewed search for a better understanding of their evolutionary history. In this study, we investigated the evolutionary diversification history of the genus *Encephalartos*, a monophyletic cycad endemic to Africa. We found an antisigmoidal pattern with a plateau and punctual explosive radiation. This pattern is typical of a constant radiation with mass extinction. The rate shift that we found may therefore be a result of a rapid recolonization of niches that have been emptied owing to mass extinction. Because the explosive radiation occurred during the transition Pliocene–Pleistocene, we argued that the processes might have been climatically mediated.

## Introduction

Current biological diversity has been shaped through macro-evolutionary processes or ecological dynamics. For example, speciation events may initially occur in bursts, owing to the availability of empty niches, and later decline as niches become occupied (Simpson 1953; Schluter 2000; Gavrilets and Vose 2005). Investigating the process of species diversification could therefore shed light on the roles ecological versus stochastic forces play in shaping species accumulation (Ricklefs 1987, 1989; Ricklefs and Schluter 1993; McPeek and Brown 2000; Stoks and McPeek 2006). Fossil records provide the best opportunity to investigate the dynamics of species accumulation, but such records are often lacking for several taxonomic groups. The phylogenetic analysis of radiation events provides an alternative tool commonly used to reconstruct the history of species diversification (Harvey et al. 1994). Such analysis of lineage splitting has revealed an emerging pattern of early-explosive radiation (Harmon et al. 2003; Shaw et al. 2003; Kadereit et al. 2004; Machordom and Macpherson 2004; Morrison et al. 2004; Williams and Reid 2004; Xiang et al. 2005; Kozak et al. 2006; Weir 2006; Phillimore and Price 2008). A pattern of increasing diversification through time is unusual as this has been showed in only very few studies (e.g., Barraclough and Vogler 2002; Linder et al. 2003; Turgeon et al. 2005).

In general, increased attention has been devoted to the geographical regions (e.g., the Cape Floristic Region) and lineages (e.g., the genus *Dianthus* L.) that are theaters of spectacular evolutionary events (Baldwin and Sanderson 1998; Richardson et al. 2001; Verboom et al. 2003; Klak et al. 2004; Kay et al. 2005; Hughes and Eastwood 2006; Garcίa-Maroto et al. 2009; Valente et al. 2010). In contrast, taxonomic groups such as gymnosperms are *a priori* of secondary interest because they are characterized by morphological stasis and low interspecific genetic variation (Van der Bank et al. 2001; Vorster 2004). However, the limited genetic variation may be indicative of recent but rapid radiations, thus raising an interesting question of what might have triggered their recent and rapid diversification.

Gymnosperms in general have long been regarded as living fossils (Hill and Brodribb 1999; Liao et al. 2004; McLoughlin and Vajda 2005; Keppel et al. 2008; Xiao et al. 2010; Álvaréz-Yepiz et al. 2011). Recent studies challenged this view (Crisp and Cook 2011; Nagalingum et al. 2011; Burleigh et al. 2012) and consequently stimulated an increased interest into the reconstruction of their evolutionary history (Crisp and Cook 2011; Burleigh et al. 2012). In an earlier study, Crisp and Cook (2009) developed a unified framework describing alternative scenarios of species diversification. These scenarios can be broadly summarized into four patterns. First, the tempo and mode of species accumulation is constant over time. This is revealed by a linear semi-log lineages-through-time (LTT) plot, indicating a constant ratio birth/death through time. Second, the pattern can depart from a linear semi-log LTT plot showing a concave or convex line as a result of single rate decrease or increase, respectively. Third, a pattern of early rapid radiation that later slows down can also be observed, driven potentially by ecological opportunities. This is generally referred to as adaptive radiation and is expected to be accompanied by the development of key innovations. The main feature of the corresponding LTT plot is an early steep slope that later flattens. Finally, it is possible that the LTT plot shows a late upswing in slope. Such pattern is generally referred to as antisigmoidal and is driven by punctual mass extinctions (Crisp and Cook 2009). On an antisigmoidal LTT plot resulting from a phylogenetic tree of only extant species, the mass extinction translates into a plateau.

In this study, we reconstructed the temporal dynamics of phylogenetic diversification of the African cycads. We also investigated how the diversification rates vary across lineages. Cycads in general include about 300 extant species in 10 genera, of which the monophyletic genus *Encephalartos* (Nagalingum et al. 2011) and its 65 species (Hill and Stevenson 2004) are endemic to Africa. Members of the genus are unequally distributed across African regions. For example, only one species occurs in West Africa (*E. barteri*) while over 50% of the *Encephalartos* species are endemic to southern Africa, a geographical region considered as the center of diversity of the genus (Golding and Hurter 2003).

Specifically, we investigated three questions: What is the net speciation rate of the genus *Encephalartos* and how does it compare with other groups? Is diversification rate constant over time? How does the diversification rate compare across lineages within the group?

## Methods

### Compilation of DNA matrix

We compiled a matrix of DNA sequences for all the 65 *Encephalartos* species. These sequences were generated in a recent phylogenetic study of the genus (Rousseau 2012). The matrix includes three plastid regions (*rbcLa*, *matK*, and *trnH-psbA*) and one nuclear region (nrITS). All voucher information and GenBank/EBI accession numbers are presented as Supplementary Information (Table S1). Also, we included in the matrix, DNA sequences of the following species that we used as outgroups and for calibration purpose: *Stangeria eriopus* (Kunze) Baill., *Macrozamia plurinervia* (L.A.S.Johnson) D.L.Jones, *Macrozamia communis* L.A.S.Johnson, *Macrozamia macdonnellii* (F.Muell. ex Miq.) A.DC., *Macrozamia pauli-guilielmi* W.Hill & F.Muell., *Lepidozamia peroffskyana* Regel, and *Lepidozamia hopei* (W.Hill) Regel.

### Tree reconstruction and estimation of divergence time

We first generated an XML file using the program BEAUTi (Bayesian Evolutionary Analysis Utility) implemented in the program BEAST (Bayesian Evolutionary Analysis by Sampling Trees; Drummond and Rambaut 2007). Then, the XML file was used to reconstruct the complete phylogeny and estimate the divergence times, using a Bayesian MCMC approach also implemented in the BEAST program. Each individual marker (*matK*, *rbcLa*, *trnH-psbA,* and nrITS) was given its own partition. We selected GTR + I + Γ as the best model of sequence evolution for each partition based on the Akaike information criterion evaluated using MODELTEST (Nylander 2004). A speciation model following a Yule process was selected as the tree prior, with an uncorrelated relaxed lognormal model for rate variation among branches. Further, we conducted simultaneous searches of topology and divergence times. For this purpose, we applied a normal prior distribution and the following secondary calibration points extracted from Nagalingum et al. (2011): *Encephalartos* crown node (11.3648 Myr), *Macrozamia* crown node (7.4836 Myr), *Lepidozamia* crown node (7.914 Myr), *Encephalartos* – *Lepidozamia* (39.7442 Myr) and (*Encephalartos* – *Lepidozamia*) – *Macrozamia* (49.037 Myr). Monte Carlo Markov Chains were run for 100 million generations with trees sampled every 10000 generations.

Log files, including prior and likelihood values, as well as the effective sample size (ESS) were examined using TRACER (Rambaut and Drummond 2007). ESS values varied between 449 and 7501 for the age estimates (Table S2), confirming stationarity. Of the resulting 10001 trees, we removed the first 2500 trees as burn-in and combined the remaining trees using TREEANNOTATOR (Rambaut and Drummond 2007) to generate a maximum clade credibility (MCC) tree. Three MCC trees were reconstructed: one based on plastid regions (*rbcLa*, *matK,* and *trnH-psbA*), one on the nuclear gene nrITS, and another tree based on the combination of all regions.

**Figure 1:**
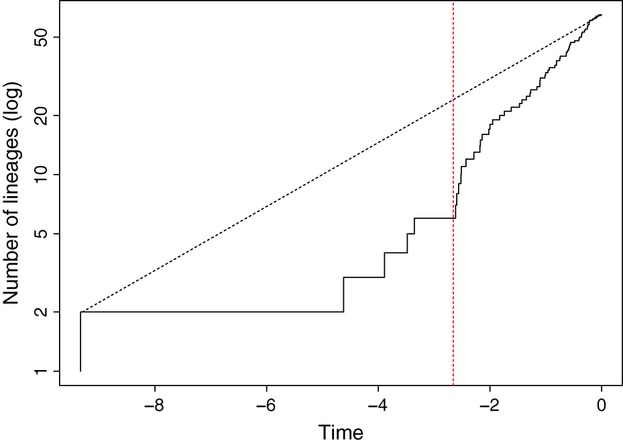
Log-lineage-through-time plot (LTT plot, full line) describing the dynamics of cladogenesis within the genus *Encephalartos* over time. The black dashed line indicates the null expectation (pure-birth model); the red dashed line indicates the period of shift in diversification around 2.6 million years before present day.

We tested for congruence between plastid and nuclear regions using the partitioned Bremer support test (DeSalle and Brower 1997) with 1000 heuristic searches, as implemented in TreeRot, version 3 (Sorenson and Franzosa 2007). A negative Bremer index is indicative of incongruence between the plastid and nuclear genes, whereas a positive score indicates congruence. Our Bremer scores were positive for all nodes, except for only one node (*E. msinganus*, *E. woodii* – *E. natalensis*). We therefore focused our statistical analyses on the MCC tree generated using the combined regions (Figs 1 & S1).

### Statistical analyses

All statistical analyses were conducted in the program R (R Core Team, 2011). First, we estimated the net diversification rate using Magallon and Sanderson's whole-clade method (Magallon and Sanderson 2001) implemented in the R library GEIGER (Harmon et al. 2008). The net rate was calculated assuming *ε* = 0 (i.e., no extinction) and *ε* = 0.9 (high extinction rate; Magallon and Sanderson 2001).

Second, we investigated the temporal patterns of clades formation, based on the lineage-through-time plot (LTT plot) and the γ-statistics of Pybus and Harvey (2000). These analyses were conducted using the R package LASER (Rabosky 2007). The value of γ describes the temporal shift in speciation along a phylogenetic tree as follows: γ < 0 indicates a pattern of decreasing speciation over time, whereas γ > 0 corresponds to acceleration in speciation toward the present day (Pybus and Harvey 2000).

Third, we conducted a model fitting analysis, testing two rate-constant models (pure-speciation and birth–death models), and four rate-variable models, including the density-dependent exponential (DDX) model, the density-dependent linear (DDL) model, the Yule2rate model and the Yule3rate model. These models were fitted under the maximum-likelihood criterion, and the best of the competing models was selected using the Akaike information criterion (AIC). We computed the ΔAIC_RC_ statistics: ΔAIC_RC_ = AIC_H0_ - AIC_H1_, where AIC_H0_ is the AIC score of the best rate-constant model and AIC_H1_ is the AIC score of the best rate-variable model (DDX, DDL, Yule2rate, or Yule3rate). This model comparison was performed using the function fitdAICrc in the R package LASER. If ΔAIC_RC_ > 0, then the best of the rate-variable models is also the best model for the observed diversification pattern; if ΔAIC_RC_ < 0, the best rate-constant model would be favored (Rabosky and Lovette 2008). The significance of the observed ΔAIC_RC_ value was tested using the function fitdAICrc.batch (also implemented in the R package LASER) with which we simulated 5000 trees of 65 tips (total number of extant *Encephalartos* species) under a pure-birth process, allowing us to generate a null distribution for ΔAIC_RC_ values.

Fourth, we tested for rate heterogeneity across lineages using the Δ_1_ statistic test of Moore et al. (2004). This test is based on the whole tree topology to detect nodes associated with significant shifts in diversification rate. The Δ_1_ statistic test was performed using the R package apTreeshape (Bortolussi et al. 2006).

Finally, as the Δ_1_ statistic test indicated a rate shift around 2.66 MYA (see Results below), we analyzed, using the γ-statistics, the pattern of temporal clade accumulation from 2.66 MYA to the present day.

## Results

We evaluated the diversification rate assuming no extinction and high extinction. We found a rate of 0.37 species per million years (sp. Myr^−1^) and 0.21 sp. Myr^−1^, respectively.

We reconstructed the LTT plot for a graphical representation of the temporal patterns of clade accumulation. Our LTT plot is best described by an antisigmoidal shape characterized by two periods of constant rate separated by a plateau (Fig. 1). The first rate constant occurs before ∼2.6 MYA, and the second after ∼2.6 MYA with a sudden rate shift at ∼2.6 MYA (Fig. 1).

**Figure 2:**
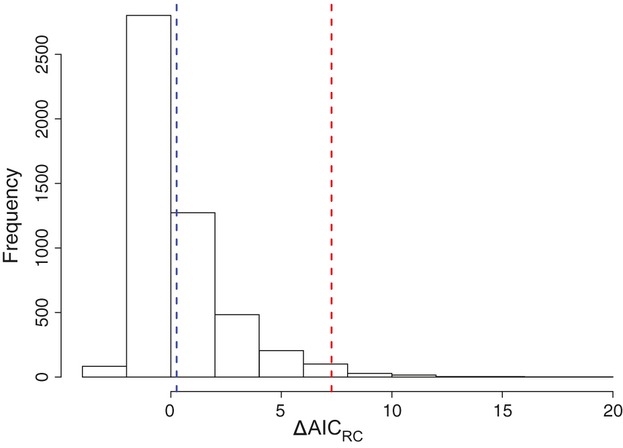
Histogram indicating the null distribution of ΔAIC_RC_. ΔAIC_RC_ is the differences between the best rate-constant model and the best rate-variable model found for 5000 simulated trees of 65 species (total number of extant *Encephalartos* species) under a Yule process; red dashed line indicates the observed value of ΔAIC_RC_, and blue dashed line indicates the mean of the null observations. The difference between observed and null is significant (*P* = 0.01).

If we ignore the punctual shift at ∼2.6 MYA, the overall diversification pattern would match that of a linear semi-log LTT plot that characterizes a constant temporal radiation. We tested this using the γ-statistics. We found a positive but nonsignificant value (γ = 0.63; *P* = 0.73), confirming an overall rate-constant diversification over time. Consequently, we would expect a rate-constant model to fit better the data. Surprisingly, our model fitting indicated that the rate-constant models were outcompeted by a rate-variable model. In particular, the Yule3rate model was favored by AIC (ΔAIC_RC_ = 7.27, *P* = 0.01; Fig. 2; Table 1). This model indicates an initial diversification rate *r*_1_ = 0.22 sp. Myr^−1^ from the origin until ∼2.66 MYA. At ∼2.66 MYA, there was a drastic shift to *r*_2_ = 3.28 sp. Myr^−1^ (almost 15 times greater than *r*_1_). However, this shift occurred only within a short period because, at 2.47 MYA, the rate decreased to *r*_3_ = 0.69 sp. Myr^−1^, which remained constant to the present day (Table 1).

**Table 1:** Results of the test for temporal variation in diversification processes of *Encephalartos*. Prior to models fitting, outgroups were removed from the tree. Models were fitted to the *Encephalartos* chronograms generated from BEAST; RC = rate-constant model; AIC = Akaike information criterion; ΔAIC_RC_ = difference in AIC scores between the best rate-constant model (pure-birth) and each of the models. The best rate-variable model is Yule3rate; *r* = net diversification rate (speciation events per million years); *a* = extinction fraction; *k* = carrying capacity; *x* = rate change parameter; st = inferred time of rate shift in million years before present

Diversification models	Log likelihood	AIC	ΔAIC_RC_	Parameters estimates		
Pure-birth (RC)	114.799	−227.598	0	*r*_1_ = 0.647	–	–
Birth–death (RC)	115.115	−226.231	−1.367	*r*_1_ = 0.520	*a* = 0.317	–
DDL	114.799	−225.598	−2	*r*_1_ = 0.647	*k* = 1065642	–
DDX	115.658	−227.317	−0.281	*r*_1_ = 0.389	*x* = 0.164	–
Yule2rate	118.831	−231.663	4.065	*r*_1_ = 0.219	*r*_2_ = 0.746; st = 2.633	
Yule3rate	122.434	−234.870	7.271	*r*_1_ = 0.22	*r*_2_ = 3.28 st_1_=2.657	*r*_3_ = 0.695 st_2_=2.474

**Figure 3:**
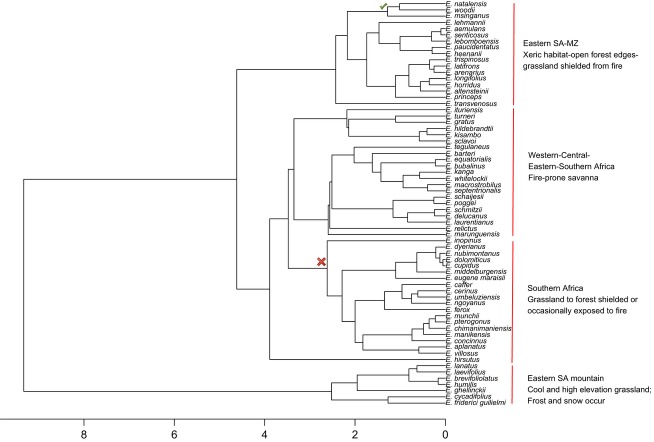
Bayesian maximum clade credibility tree of *Encephalartos* inferred from the combination of all four DNA regions included in this study. Outgroups are not shown (but see Figure S1). The red cross indicates the node of the southern African clade with highest diversification rate. The geographical pattern of species along the phylogeny is indicated as well as habitat preferences. The green “tick” symbol indicates the node where nuclear and plastid genes were incongruent. Dates on the scale axis are in million years. SA = South Africa; MZ = Mozambique.

Furthermore, we investigated whether the 15-time rate shift that occurred around 2.66 MYA was significant compared with the rate across all nodes in the phylogeny. Our analysis using the Δ_1_ statistic test gave support to this, indicating a significant shift in diversification rate at the node corresponding to a southern African lineage (*P* = 0.015; Fig. 3).

Finally, we examined the patterns of clades accumulation from 2.66 MYA to the present day using the γ-statistics. We found a negative but still nonsignificant value (γ = −0.80, *P* = 0.21), suggesting that, in addition to the rate-constant diversification before ∼2.66 MYA, the diversification from ∼2.66 MYA onward was also constant. Overall, the diversification rate of the African cycad is constant before and after ∼2.66 MYA with a drastic increase around 2.66 MYA.

## Discussion

Our estimate of absolute net diversification rate for the genus *Encephalartos* assuming no extinction (0.372 sp. Myr^−1^) is comparable with that of the angiosperms under similar assumption, but only for the angiosperms younger than 30 Myr (0.349 sp. Myr^−1^; Crisp and Cook 2011). However, this rate is greater than the mean rate found for all gymnosperms in general (0.166 sp. Myr^−1^; Crisp and Cook 2011). Conversely, under the assumption of high extinction rate, our net rate for *Encephalartos* (0.208 sp. Myr^−1^) is lower than that of the angiosperms (1.713 sp. Myr^−1^) and gymnosperms (0.721 sp. Myr^−1^; Crisp and Cook 2011). Also, our rate is far lower than those reported for other plant taxonomic groups especially angiosperms that underwent spectacular radiation (e.g., see Baldwin and Sanderson 1998; Richardson et al. 2001; Verboom et al. 2003; Klak et al. 2004; Kay et al. 2005; Hughes and Eastwood 2006; Garcίa-Maroto et al. 2009; Valente et al. 2010). This lower speed of diversification may arise due to a high background rate of extinction, lower rate of speciation, or the combination thereof (Hill and Brodribb 1999; Cantrill and Poole 2005; Ricklefs 2006; Mittelbach et al. 2007; Schemske 2009; Gorelick and Olson 2011). A recent study revealed that a high extinction rate in gymnosperms is more likely specifically in a recent past (7–5 MYA; Niklas 1997; Crepet and Niklas 2009); we propose here that this past extinction may also account for the low rate of diversification found in this study for *Encephalartos*.

Looking into the overall diversification over time, we found evidence for a rate-constant pattern (γ-statistics). Counterintuitively, the AIC statistics favored a Yule3rate model over both rate-constant models tested. Why these apparently conflicting results? From the origin to 2.66 MYA, the diversification rate was constant. The rate was also constant from 2.66 MYA to the present day. However, at 2.66 MYA, a significant rate shift occurred, but this rate decreased almost immediately to a lower rate that remained constant toward the present day. Because the acceleration and deceleration occurred almost immediately, both events might offset their respective effects on the overall patterns of speciation. As a result, the punctual rate shift observed does not influence significantly the overall diversification rate. However, it creates a pattern of rate heterogeneity across lineages, as indicated by the fit of a rate-variable model.

Alternative hypotheses underlying the rate shift include meta-community dynamics (McPeek 2008) and acceleration of molecular evolution (Bromham 2003). However, adaptive radiation with the development of key innovations (Klak et al. 2004; Ree 2005) perhaps driven by environmental change (Lovette and Bermingham 1999) has for long been the emerging explanation (see reviews in Gavrilets and Losos 2009; Glor 2010), and this was found in diverse biological systems including lizards (Harmon et al. 2003), birds (Weir 2006; Phillimore and Price 2008), fish (Rüber and Zardoya 2005), and plants (Davies et al. 2004). Indeed, the punctual explosive radiation that we found coincides with the environmental shift from the Pliocene epoch (5.3–2.6 MYA) where climate was cooler, drier (compared with the immediately preceding Miocene), and seasonal to a new climatic regime in the Pleistocene (2.6 MYA–11.700 MYA) characterized by repeated glaciation events worldwide. During that period of global glaciation events, the African climate shifted to more arid conditions (deMenocal 1995), especially in subtropical Africa (deMenocal 2004). The increased aridification may have promoted the radiation of species capable of surviving the changing environment through a development of key innovations. The occurrence of subterranean stems in the southern African clade that underwent the punctual explosive radiation (Fig. 3) may be regarded, to some extent, as key innovation developed to adapt to high temperature and general aridity that prevailed during the Pliocene–Pleistocene transition in southern Africa. Members of this clade occur predominantly at cool, high elevations in gorges and rocks, but also in fire-prone ecosystems (savannas, grasslands) and forests occasionally exposed to fire (Vorster 2004). This adds to the ongoing controversy surrounding the role of the Quaternary climatic change as a driver of speciation; evidence of Quaternary speciation has been reported in some analyses (e.g., Weir and Schluter 2007; Janssens et al. 2009; Valente et al. 2010; Mullen et al. 2011; Nagalingum et al. 2011), but not in others (e.g., Hoorn et al. 2010; Wesselingh et al. 2010).

However, there are a number of evidences that discount these alternative hypotheses including explosive radiation as the strong forces shaping the observed antisigmoidal pattern. First, typical explosive radiation results in an increase without plateau (Turgeon et al. 2005; McKenna & Farrell, 2006). Second, the fact that six different genera of cycads diverge almost simultaneously across the globe (Nagalingum et al. 2011) underscore the development of innovative characters as underlying the synchronous diversification globally. Third, the phylogeny of the African cycads is characterized by “phylogenetic fuses,” indicated by long branches from the origin (∼9 MYA) toward the tips (the last 1 MYA) where the largest radiation of the genus occurs (Fig. 3). Such phylogenetic fuses were also reported, for example, by Nagalingum et al. (2011) for all cycads in general and could be either the result of low diversification or mass extinction. Combining simulations and empirical data, Crisp and Cook (2009) demonstrated convincingly that the pattern of antisigmoidal curve with a plateau is driven by mass extinctions. Indeed, gymnosperms in general experienced a mass extinction between 7–5 Myr (Niklas 1997; Crepet and Niklas 2009), a timeframe preceding roughly the period of large radiation of the African cycads (see Fig. 3). As such, the observed explosive radiation may be the result of a rapid recolonization of niches that have been emptied during the mass extinction events.

Overall, the diversification of the African cycads is constant through time. However, the mass extinction that occurred between 7 and 5 Myr (Niklas 1997; Crepet and Niklas 2009) may have created empty niches that have been refilled during the transition Pliocene–Pleistocene, leading to the accelerated radiation observed. The standing diversity of cycads on African continent may therefore have been shaped by the Quaternary climatic change.

## Supporting Information

**Figure S1.** Bayesian maximum clade credibility chronogram inferred from the combined plastid + nuclear data (*rbcLa + matK + trnH-psbA* + nrITS) with 95% highest posterior density for node ages (blue boxes along branches).

**Table S1.** List of samples included in this study and their GenBank/EBI accession numbers.

**Table S2.** Summary statistics of TRACER analysis.
