# Supplementary figures and images for: Evidence of constant diversification punctuated by a mass extinction in the African cycads
Source: Ecol Evol. 2013 Dec 11;4(1):50–8. doi: 10.1002/ece3.880 (PMC3894887; doi:10.1002/ece3.880)

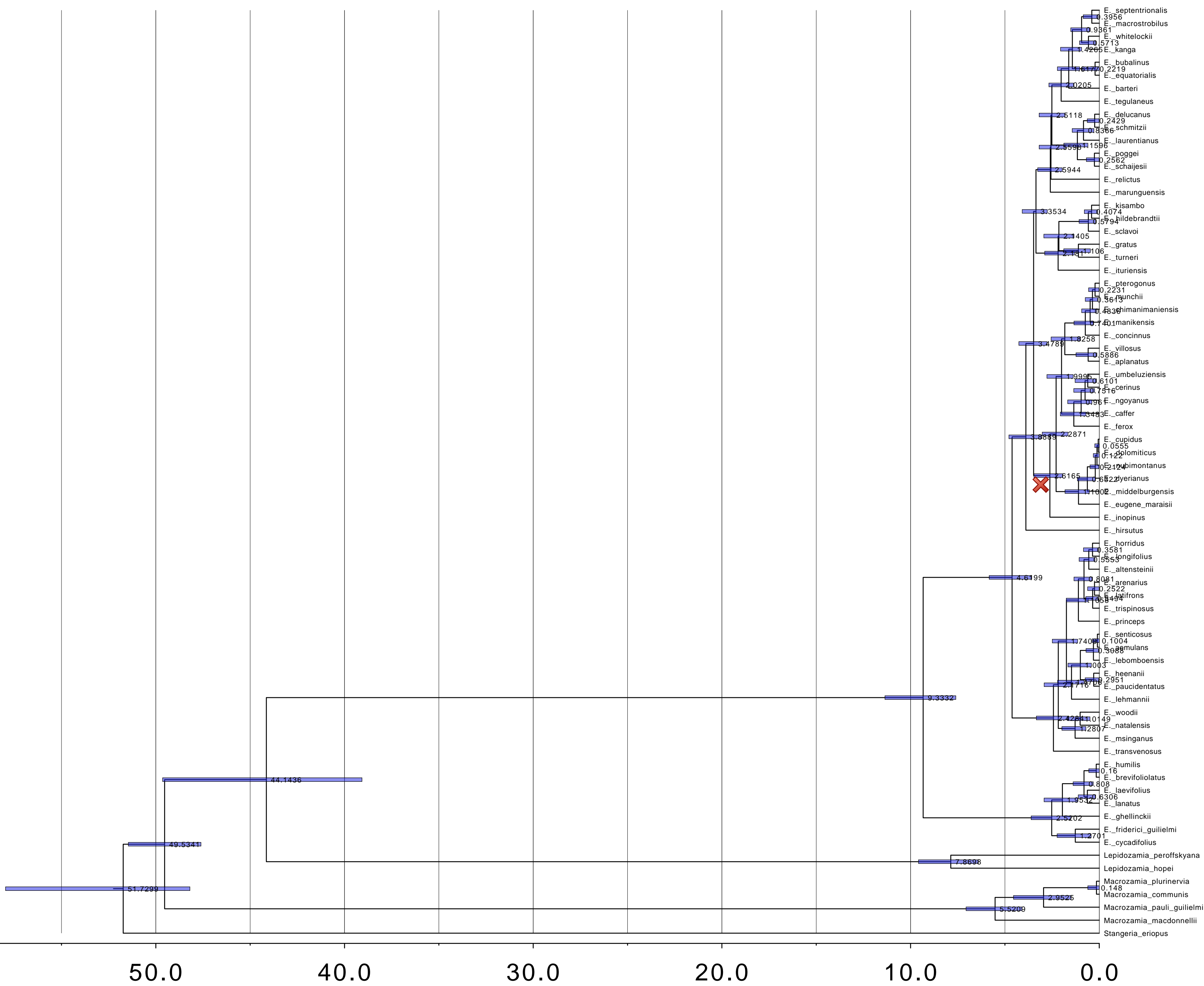

Supplement: Supplementary file 1 [file ece30004-0050-SD1.pdf]
