# Supplementary material for: Evidence of constant diversification punctuated by a mass extinction in the African cycads
Source: Ecol Evol. 2013 Dec 11;4(1):50–8. doi: 10.1002/ece3.880 (PMC3894887; doi:10.1002/ece3.880)
Supplement: Supplementary file 2 [file ece30004-0050-SD2.doc]

**Table S1.** List of samples included in this study. Abbreviations for collection sources: Private Collection 1 (PC1), Private collection 2 (PC2), Private collection 3 (PC3), University of Johannesburg (UJ), University of Pretoria Experimental farm (EF), University of Pretoria Manie van der Schijff Botanical Garden (MBG), Cycad World of Innovation (CWOFI), Lowveld Botanical Garden (LBG), New York Botanical Garden DNA bank (NYBG), Pretoria Botanical Garden (PRE), Umtamvuna Nature Reserve (UNR), Rhenosterpoort Nature Reserve (RNR), Xander de Kock Donation (XDK), Middelburg cycad trail (MCT). – indicates missing BOLD or GenBank accession numbers; *E., Encephalartos*

| **Species** | **Collection Number** | | | | | **Source** | **Origin** | | **Form name** | | **BOLD Accession** | | | **GenBank Accession** | | | | | | |
| --- | --- | --- | --- | --- | --- | --- | --- | --- | --- | --- | --- | --- | --- | --- | --- | --- | --- | --- | --- | --- |
|  |  | | | | |  |  | |  | |  | | | ***rbc*La** | ***mat*K** | | ***psb*A-*trn*H** | | **nrITS** | |
| **Samples used in analyses** | | | | | | | | | | |  | | |  |  | |  | |  | |
| *E*. *aemulans* Vorster | | | *PR861* JRAU | | | MBG | Wild | |  | | CYPAF309 | | | JQ025439 | JQ046261 | | JQ045891 | | JQ046123 | |
|  | | |  | | |  |  | |  | |  | | |  |  | |  | |  | |
| *E*. *altensteinii* Lehm. | | | *d1019* NY | | | NYBG | Wild | |  | | - | | | - | - | | - | | - | |
| *E*. *aplanatus* Vorster | | | *PR682* JRAU | | | PRE | Wild | |  | | CYAF033 | | | JQ025446 | JQ046259 | | JQ045969 | | JQ046121 | |
|  | | |  | | |  |  | |  | |  | | |  |  | |  | |  | |
| *E*. *arenarius* R.A.Dyer | | | *PR758* JRAU | | | CWOFI | Cultivated | | Alexandria | | CYAF104 | | | JQ025453 | JQ046258 | | JQ045978 | | JQ046120 | |
| *E. barteri* | | | *PR892 JRAU* | | | EF | Cultivated | |  | |  | | | JQ025458 |  | |  | | JQ046117 | |
|  | | |  | | |  |  | |  | |  | | |  |  | |  | |  | |
| *E*. *brevifoliolatus* Vorster | | | *Xdk 1* JRAU | | | XDK | Wild | | Wolkeberg | | CYPAF378 | | | JQ025460 | JQ046254 | | JQ045976 | | JQ046116 | |
|  | | |  | | |  |  | |  | |  | | |  |  | |  | |  | |
| *E*. *bubalinus* Melville | | | *PR885* JRAU | | | EF | Cultivated | |  | | CYPAF333 | | | JQ025466 | JQ046252 | | JQ045974 | | JQ046114 | |
|  | | |  | | |  |  | |  | |  | | |  |  | |  | |  | |
|  | | |  | | |  |  | |  | |  | | |  |  | |  | |  | |
| *E*. *caffer* (Thunb.) Lehm. | | | *PR729* JRAU | | | PC 2 | Cultivated | |  | | CYAF076 | | | JQ025468 | JQ046250 | | JQ045972 | | JQ046112 | |
|  | | |  | | |  |  | |  | |  | | |  |  | |  | |  | |
| *E*. *cerinus* Lavranos & D.L.Goode | | | *PR859* JRAU | | | MBG | Wild | |  | | CYPAF307 | | | JQ025475 | JQ046249 | | JQ045971 | | JQ046111 | |
|  | | |  | | |  |  | |  | |  | | |  |  | |  | |  | |
| *E*. *chimanimaniensis* R.A.Dyer & Verdoorn | | | *PR883* JRAU | | | LBG | Cultivated | |  | | CYPAF331 | | | JQ025477 | JQ046248 | | JQ045970 | | JQ046110 | |
|  | | |  | | |  |  | |  | |  | | |  |  | |  | |  | |
| *E*. *concinnus* R.A.Dyer & Verdoorn | | | *PR890* JRAU | | | EF | Cultivated | |  | | CYPAF338 | | | JQ025479 | JQ046246 | | JQ045968 | | JQ046108 | |
|  | | |  | | |  |  | |  | |  | | |  |  | |  | |  | |
| *E*. *cupidus* R.A.Dyer | | | *PR691* JRAU | | | PC 1 | Cultivated | |  | | CYAF042 | | | JQ025481 | JQ046245 | | JQ045967 | | JQ046107 | |
|  | | |  | | |  |  | |  | |  | | |  |  | |  | |  | |
| *E*. *cycadifolius* (Jacq.) Lehm. | | | *PR683* JRAU | | | PRE | Wild | |  | | CYAF034 | | | JQ025483 | JQ046243 | | JQ045965 | | JQ046105 | |
| *E. delucanus* | | | *d1129 NY* | | | NYBG | Wild | |  | | - | | | - | - | | - | | - | |
| *E*. *dolomiticus* Lavranos & D.L.Goode | | | *PR865* JRAU | | | MBG | Wild | |  | | CYPAF313 | | | JQ025489 | JQ046242 | | JQ045964 | | JQ046104 | |
|  | | |  | | |  |  | |  | |  | | |  |  | |  | |  | |
| *E*. *dyerianus* Lavranos & D.L.Goode | | | *PR731* JRAU | | | PC 2 | Cultivated | |  | | CYAF078 | | | JQ025491 | JQ046241 | | JQ045963 | | JQ046103 | |
|  | | |  | | |  |  | |  | |  | | |  |  | |  | |  | |
| *E*. *equatorialis* P.J.H.Hurter | | | *PR900* JRAU | | | EF | Cultivated | |  | | CYPAF348 | | | JQ025494 | JQ046239 | | JQ045961 | | JQ046101 | |
| *E*. *eugene-maraisii* Verd. | | | *PR872* JRAU | | | MBG | Wild | |  | | CYPAF320 | | | JQ025502 | JQ046238 | | JQ045960 | | JQ046100 | |
|  | | |  | | |  |  | |  | |  | | |  |  | |  | |  | |
| *E*. *ferox* G.Bertol. | | | *PR771* JRAU | | | CWOFI | Cultivated | |  | | CYAF116 | | | JQ025505 | JQ046301 | | JQ046017 | | JQ046156 | |
| *E*. *friderici-guilielmi* Lehm. | | | *PR772* JRAU | | | CWOFI | Cultivated | |  | | CYAF117 | | | JQ025514 | JQ046235 | | JQ045957 | | JQ046097 | |
|  | | |  | | |  |  | |  | |  | | |  |  | |  | |  | |
| *E*. *ghellinckii* Lem. | | | *PR773* JRAU | | | CWOFI | Cultivated | |  | | CYAF118 | | | JQ025518 | JQ046232 | | JQ045954 | | JQ046094 | |
|  | | |  | | |  |  | |  | |  | | |  |  | |  | |  | |
| *E*. *gratus* Prain | | | *PR774* JRAU | | | CWOFI | Cultivated | |  | | CYAF119 | | | JQ025520 | JQ046231 | | JQ045953 | | JQ046093 | |
|  | | |  | | |  |  | |  | |  | | |  |  | |  | |  | |
| *E*. *heenanii* R.A.Dyer | | | *PR775* JRAU | | | CWOFI | Cultivated | | Short leaf | | CYAF120 | | | JQ025528 | JQ046229 | | JQ045951 | | JQ046091 | |
|  | | |  | | |  |  | |  | |  | | |  |  | |  | |  | |
| *E*. *hildebrandtii* A.Braun & Bouché | | | *PR824* JRAU | | | CWOFI | Cultivated | |  | | CYAF169 | | | JQ025529 | JQ046227 | | JQ045949 | | JQ046089 | |
|  | | |  | | |  |  | |  | |  | | |  |  | |  | |  | |
| *E*. *hirsutus* P.J.H.Hurter | | | *PR718* JRAU | | | PC 2 | Cultivated | |  | | CYAF069 | | | JQ025534 | JQ046226 | | JQ045948 | | JQ046088 | |
|  | | |  | | |  |  | |  | |  | | |  |  | |  | |  | |
|  | | |  | | |  |  | |  | |  | | |  |  | |  | |  | |
|  | | |  | | |  |  | |  | |  | | |  |  | |  | |  | |
|  | | |  | | |  |  | |  | |  | | |  |  | |  | |  | |
| *E*. *horridus* (Jacq.) Lehm. | | | *PR777* JRAU | | | CWOFI | Cultivated | |  | | CYAF122 | | | JQ025536 | JQ046299 | | JQ046015 | | JQ046154 | |
| *E*. *humilis* Verd. | | | *PR712* JRAU | | | PC 2 | Cultivated | |  | | CYAF063 | | | JQ025541 | - | | - | | - | |
|  | | |  | | |  |  | |  | |  | | |  |  | |  | |  | |
| *E*. *inopinus* R.A.Dyer | | | *PR778* JRAU | | | CWOFI | Cultivated | |  | | CYAF123 | | | JQ025546 | JQ046222 | | JQ045944 | | JQ046084 | |
| *E. ituriensis* | | | *d1131 NY* | | | NYBG | Wild | |  | | - | | | - | - | | - | | - | |
| *E*. *kanga* Pócs & Q.Luke | | | *PR907* JRAU | | | EF | Cultivated | |  | | CYPAF355 | | | JQ025658 | JQ046298 | | JQ046014 | | JQ046153 | |
| *E*. *kisambo* Faden & Beentje | | | *PR745* JRAU | | | PC 2 | Cultivated | |  | | CYAF091 | | | JQ025551 | JQ046220 | | JQ045942 | | JQ046082 | |
|  | | |  | | |  |  | |  | |  | | |  |  | |  | |  | |
|  | | |  | | |  |  | |  | |  | | |  |  | |  | |  | |
|  | | |  | | |  |  | |  | |  | | |  |  | |  | |  | |
| *E*. *laevifolius* Stapf & Burtt Davy | | | *PR803* JRAU | | | CWOFI | Cultivated | | Mallalosha | | CYAF148 | | | JQ025552 | JQ046216 | | JQ045938 | | JQ046078 | |
|  | | |  | | |  |  | |  | |  | | |  |  | |  | |  | |
|  | | |  | | |  |  | |  | |  | | |  |  | |  | |  | |
| *E*. *lanatus* Stapf & Burtt Davy | | | *d1133* NY | | | NYBG | Wild | |  | | - | | | - | - | | - | | - | |
|  | | |  | | |  |  | |  | |  | | |  |  | |  | |  | |
| *E. latifrons* | | | *PR678 JRAU* | | | PBG | Wild | |  | |  | | | JQ025568 | JQ046212 | | JQ045934 | | JQ046074 | |
|  | | |  | | |  |  | |  | |  | | |  |  | |  | |  | |
| *E*. *laurentianus* De Wild. | | | *d1029* NY | | | NYBG | Wild | |  | | - | | | - | - | | - | | - | |
| *E*. *lebomboensis* Verd. | | | *PR657* JRAU | | | UJ | Cultivated | | Piet Retief | | CYAF008 | | | JQ025576 | JQ046209 | | JQ045931 | | JQ046071 | |
|  | | |  | | |  |  | |  | |  | | |  |  | |  | |  | |
| *E*. *lebomboensis* Verd. | | | *PR874* JRAU | | | MBG | Wild | |  | | CYPAF322 | | | JQ025574 | JQ046292 | | JQ046008 | | JQ046147 | |
| *E*. *lehmannii* Lehm. | | | *PR661* JRAU | | | PRE | Wild | |  | | CYAF012 | | | JQ025585 | JQ046206 | | JQ045928 | | JQ046068 | |
|  | | |  | | |  |  | |  | |  | | |  |  | |  | |  | |
| *E*. *longifolius* (Jacq.) Lehm. | | | *PR809* JRAU | | | CWOFI | Cultivated | | Blunt Tip | | CYAF154 | | | JQ025591 | JQ046204 | | JQ045926 | | JQ046066 | |
|  | | |  | | |  |  | |  | |  | | |  |  | |  | |  | |
| *E. macrostrobilus* | | | *CC333 NY* | | | NYBG | Wild | |  | | - | | | - | - | | - | | - | |
|  | | |  | | |  |  | |  | |  | | |  |  | |  | |  | |
| *E*. *manikensis* (Gilliland) Gilliland | | | *PR903* JRAU | | | EF | Cultivated | | Vumba | | CYPAF351 | | | JQ025597 | JQ046201 | | JQ045923 | | JQ046063 | |
|  | | |  | | |  |  | |  | |  | | |  |  | |  | |  | |
| *E*. *marunguensis* Devred | | | *PR912* JRAU | | | EF | Cultivated | |  | | CYPAF360 | | | JQ025602 | JQ046200 | | JQ045922 | | JQ046062 | |
|  | | |  | | |  |  | |  | |  | | |  |  | |  | |  | |
| *E*. *middelburgensis* Vorster, Robbertse & S.van der Westh. | | | *PR827* JRAU | | | MCT | Wild | |  | | CYAF172 | | | JQ025605 | JQ046287 | | JQ046003 | | JQ046143 | |
|  | | |  | | |  |  | |  | |  | | |  |  | |  | |  | |
| *E*. *msinganus* Vorster | | | *PR701* JRAU | | | PC 1 | Cultivated | |  | | CYAF052 | | | JQ025610 | JQ046198 | | JQ045919 | | JQ046059 | |
|  | |  | |  |  | | |  | |  | |  |  | | |  | |  | |  |
| *E*. *munchii* R.A.Dyer & Verdoorn | | | *PR737* JRAU | | | PC 2 | Cultivated | |  | | CYAF083 | | | JQ025614 | JQ046196 | | JQ045917 | | JQ046057 | |
|  | | |  | | |  |  | |  | |  | | |  |  | |  | |  | |
|  | | |  | | |  |  | |  | |  | | |  |  | |  | |  | |
|  | | |  | | |  |  | |  | |  | | |  |  | |  | |  | |
| *E*. *natalensis* R.A.Dyer & Verdoorn | | | *d1035* NY | | | NYBG | Wild | |  | | - | | | - | - | | - | | - | |
|  | | |  | | |  |  | |  | |  | | |  |  | |  | |  | |
| *E*. *ngoyanus* Verd*.* | | | *PR717* JRAU | | | PC 2 | Cultivated | |  | | CYAF068 | | | JQ025626 | JQ046193 | | JQ045914 | | JQ046054 | |
|  | | |  | | |  |  | |  | |  | | |  |  | |  | |  | |
| *E*. *nubimontanus* P.J.H.Hurter | | | *PR655* JRAU JRAU | | | UJ | Cultivated | |  | | CYAF006 | | | JQ025631 | JQ046191 | | JQ045912 | | JQ046052 | |
|  | | |  | | |  |  | |  | |  | | |  |  | |  | |  | |
| *E*. *paucidentatus* Stapf & urtt Davy | | | *PR710* JRAU | | | PC 2 | Cultivated | |  | | CYAF061 | | | JQ025632 | JQ046188 | | JQ045909 | | JQ046049 | |
|  | | |  | | |  |  | |  | |  | | |  |  | |  | |  | |
| *E*. *poggei* Asch. | | | *PR813* JRAU | | | CWOFI | Wild | |  | | CYAF158 | | | JQ025638 | JQ046187 | | JQ045908 | | JQ046048 | |
|  | | |  | | |  |  | |  | |  | | |  |  | |  | |  | |
| *E*. *princeps* R.A.Dyer | | | *PR810* JRAU | | | CWOFI | Cultivated | |  | | CYAF155 | | | JQ025641 | JQ046186 | | JQ045907 | | JQ046047 | |
|  | | |  | | |  |  | |  | |  | | |  |  | |  | |  | |
| *E*. *pterogonus* R.A.Dyer & Verdoorn | | | *PR876* JRAU | | | PC 1 | Cultivated | |  | | CYPAF324 | | | JQ025642 | JQ046184 | | JQ045905 | | JQ046045 | |
| *E. schaijesii* | | | *CC447 NY* | | | NYBG | Wild | |  | | - | | | - | - | | - | | - | |
| *E*. *schmitzii* Malaisse | | | *PR819* JRAU | | | CWOFI | Cultivated | |  | | CYAF164 | | | JQ025644 | JQ046183 | | JQ045904 | | JQ046044 | |
|  | | |  | | |  |  | |  | |  | | |  |  | |  | |  | |
| *E*. *sclavoi* De Luca, D.W.Stev. & A.Moretti | | | *PR738* JRAU | | | PC 2 | Cultivated | |  | | CYAF084 | | | JQ025647 | JQ046280 | | JQ045996 | | JQ046136 | |
|  | | |  | | |  |  | |  | |  | | |  |  | |  | |  | |
| *E*. *senticosus* Vorster | | | *PR663* JRAU | | | PRE | Wild | |  | | CYAF014 | | | JQ025650 | JQ046182 | | JQ045903 | | JQ046043 | |
|  | | |  | | |  |  | |  | |  | | |  |  | |  | |  | |
| *E*. *septentrionalis* | | | *d1138 NY* | | | NYBG | - | | - | | - | | | - | - | | - | | - | |
| *E*. *sp*. | | | *PR746* JRAU | | | PC 2 | Cultivated | |  | | CYAF092 | | | - | - | | - | | - | |
| *E*. *tegulaneus* Melville | | | *PR825* JRAU | | | CWOFI | Cultivated | |  | | CYAF170 | | | JQ025665 | - | | JQ046029 | | JQ046168 | |
|  | | |  | | |  |  | |  | |  | | |  |  | |  | |  | |
| *E*. *transvenosus* Stapf & Burtt Davy | | | *PR727* JRAU | | | PC 2 | Cultivated | |  | | CYAF074 | | | JQ025671 | JQ046179 | | JQ045900 | | JQ046040 | |
|  | | |  | | |  |  | |  | |  | | |  |  | |  | |  | |
|  | | |  | | |  |  | |  | |  | | |  |  | |  | |  | |
| *E*. *trispinosus* (Hook.) R.A.Dyer | | | *PR868* JRAU | | | MBG | Wild | |  | | CYPAF316 | | | JQ025674 | JQ046177 | | JQ045898 | | JQ046038 | |
|  | | |  | | |  |  | |  | |  | | |  |  | |  | |  | |
|  | | |  | | |  |  | |  | |  | | |  |  | |  | |  | |
| *E*. *turneri* Lavranos & D.L.Goode | | | *d1044* NY | | | NYBG | Wild | |  | | - | | | - | - | | - | | - | |
|  | | |  | | |  |  | |  | |  | | |  |  | |  | |  | |
| *E*. *umbeluziensis* R.A.Dyer | | | *d1046* NY | | | NYBG | Wild | |  | | - | | | - | - | | - | | - | |
| *E*. *villosus* Lem. | | | *PR671* JRAU | | | PRE | Wild | |  | | CYAF022 | | | JQ025694 | JQ046173 | | JQ045894 | | JQ046034 | |
|  | | |  | | |  |  | |  | |  | | |  |  | |  | |  | |
| *E*. *whitelockii* P.J.H.Hurter | | | *d1048* NY | | | NYBG | Wild | |  | | - | | | - | - | | - | | - | |
|  | | |  | | |  |  | |  | |  | | |  |  | |  | |  | |
| *E*. *woodii* Sander | | | *PR675* JRAU | | | PRE | Wild | |  | | CYAF026 | | | JQ025702 | JQ046170 | | - | | JQ046031 | |
|  | | |  | | |  |  | |  | |  | | |  |  | |  | |  | |
